# Assessing the Influence of Electrode Polarity on the Treatment of Poultry Slaughterhouse Wastewater

**DOI:** 10.3390/molecules27031014

**Published:** 2022-02-02

**Authors:** Kulyash Meiramkulova, Tursynkul Bazarbayeva, Raigul Orynbassar, Almas Tleukulov, Nabiollina Madina, Togzhan Mashan, Akubayeva Dariya, Ainagul Apendina, Nurgul Nurmukhanbetova

**Affiliations:** 1 Department of Environmental Engineering and Management, L.N. Gumilyov Eurasian National University, Satpayev Street 2, Nur-Sultan 010000, Kazakhstan; 2 Department of the UNESCO Chair for Sustainable Development, Al-Farabi Kazakh National University, Al-Farabi 71, Almaty 050040, Kazakhstan; tursynkul.bazarbayeva@gmail.com (T.B.); a_dariya@mail.ru (A.D.); 3 Department of Physical Chemistry, Catalysis and Oil Chemistry, Al-Farabi Kazakh National University, Al-Farabi 71, Almaty 050040, Kazakhstan; raihan_06_79@mail.ru; 4 Department of Water Resources and Reclamation, Kazakh National Agrarian University, Abaya Ave. 8, Almaty 050000, Kazakhstan; almas58@mail.ru (A.T.); nabiollina73@mail.ru (N.M.); 5 Department of Chemistry, L.N. Gumilyov Eurasian National University, Satpayev Street 2, Nur-Sultan 010000, Kazakhstan; togzhgan-mashan@mail.ru; 6 Department of Chemistry and Chemical Technology, K. Zhubanov Aktobe Regional University, A. Moldagulovoi Ave. 34, Aktobe 030000, Kazakhstan; k.ajnagul@mail.ru; 7 Department of Chemistry and Biotechnology, Sh. Ualikhanov Kokshetau University, Kokshetau City, Abai Str. 76, Nur-Sultan 020000, Kazakhstan; nn_nurgul@mail.ru

**Keywords:** electrochemical wastewater treatment, water quality, electrode polarity, physicochemical pollutants, electrode material

## Abstract

Electrochemical methods have been increasingly gaining popularity in the field of wastewater treatment. However, the performance of these methods can be highly affected by the polarity direction as determined by the electrodes arrangement (anode to cathode or cathode to anode); as well as the characteristics of the wastewater to be treated as determined by the type of wastewater. The presented research work investigated the relationship between polarity direction and the removal of pollutants from poultry slaughterhouse wastewater using titanium and aluminium electrode materials. In the first case, the wastewater was exposed to the Ti (anode)-Al (cathode) combination, whereas in the second case the wastewater was subjected to the Al (anode)-Ti (cathode) arrangement. The two cases were designed to see if the polarity direction of the chosen electrode materials affected the removal of pollutants. The removal efficiencies were computed as a ratio of the remaining concentration in the treated effluent to the concentration before treatment. It was observed that the production processes generate highly fluctuating wastewater in terms of pollution loading; for instance, 422 to 5340 Pt-Co (minimum to maximum) were recorded from color, 126 to 2264 mg/L were recorded from total dissolved solids, and 358 to 5998 mg/L from chemical oxygen demand. Also, the research results after 40 min of retention time showed that both electrode arrangements achieved relatively high removal efficiencies; Whereby, the aluminium to titanium polarity achieved up to 100% removal efficiency from turbidity while the titanium to aluminium polarity achieved a maximum of 99.95% removal efficiency from turbidty. Also, a similar phenomenon was observed from total dissolved solids; whereby, on average 0 mg/L was achieved when the wastewater was purified using the aluminium to titanium arrangement, while on average 2 mg/L was achieved from the titanium to aluminium arrangement. A little higher removal efficiency discrepancy was observed from ammonia; whereby, the aluminium to titanium arrangement outperformed the titanium to aluminium arrangement with average removal efficiencies of 82.27% and 64.11%, respectively.

## 1. Introduction

Poultry production activities are among the giant water consumers and wastewater generators in the world. The phenomenon is expected to be continuously growing due to the fact that, the demand for poultry products has also been rapidly increasing as a result of the high population growth worldwide [1]. It is estimated that the poultry production processes generate approximately 20 to 40 L of wastewater per processed bird, with 25 L being a typical value [2]. It is also worth noting that, poultry slaughterhouse serves as the crucial unit in the poultry production processes [3]. The main sections in the poultry slaughterhouse are; defeathering, evisceration, and cooling. The most challenging part is that the activities in the slaughterhouse generate one of the highly polluted types of wastewaters [4]. More specifically, the generated wastewater is characterized by a high level of biochemical oxygen demand (BOD), chemical oxygen demand (COD), total suspended solids (TSS), and a complex mixture of fats, proteins, and fibers requiring systematic treatment before discharge or recycling [5,6].

Although there are already many technologies used in the field of wastewater treatment in general [7,8,9,10,11], however, the performance of these systems is highly dependent on the type of technology used and characteristics of the wastewater to be treated. Generally, poultry slaughterhouse wastewater can be purified using physical [12], chemical [13], and biological-based [14] treatment approaches. But it has to be noted that, each treatment technology has its strengths (advantages) and weaknesses (disadvantages). For instance, although the biological treatment systems, both anaerobic and aerobic are relatively strong in terms of the microorganism’s adaptability to a wide variety of wastewater composition; however, these systems are relatively slow treatment processes requiring large physical areas while generating huge volumes of sludge [15,16]. This is because biological wastewater treatment relies on microorganisms to assimilate organic matter and nutrients present in the wastewater [17]. However, these microorganisms need time to properly digest the pollutants in the wastewater [18]. In general, the biological treatment processes are the most widely applicable technologies for poultry slaughterhouse wastewater. In the literature, several investigations based on up-flow anaerobic sludge blanket reactors achieved more than 90% COD removal efficiency from poultry slaughterhouse wastewater [19,20]. According to Aziz et al., that investigated the performance of submerged fibers in an attached growth sequential batch reactor for poultry slaughterhouse wastewater treatment, more than 93% for BOD and COD was achieved,

The physical treatment processes such as membrane filtration (MF) systems including reverse osmosis are among the most highly efficient treatment approaches in terms of pollutant removal, but they are giant power consumers as to operate the system high pressure is needed [21]. Also, they are well-known in terms of sludge generation and the generated sludge has to be handled separately. The whole process makes the treatment process relatively expensive and less feasible for large-scale treatments and low-income communities [22]. On the other hand, there are also chemical-based treatment technologies such as electrochemical (EC) systems that act as a potential alternative to the poultry slaughterhouse wastewater treatment [23,24,25]. The EC treatment systems offer a number of advantages such as being robust, requiring a relatively small working area, as well as being relatively strong and flexible under fluctuating wastewater flows and composition. In general, the EC treatment technologies have been of recent highly gaining researchers’ interest in the field of wastewater treatment, for example; Sharma Swati and Simsek Halis [26] investigated the applicability of EC methods for the treatment of sugar beet industry process wastewater, Davarnejad Reza and Nikseresht Mehrazin [27] for diary wastewater, Hoang Tran Le and Luu Tran Le [28] for textile wastewater as well as Tien Tran Tan and Le Luu Tran [29] for tannery wastewater.

The general working mechanism of an EC treatment system depends on at least two electrodes (an anode and a cathode), and also there should be an intermediate space filled with electrolytes [30]. It is also important to understand that, the terms anode and cathode are not fixed and are highly dependent on the direction of current passing through the electrode rather than the voltage polarity of the particular electrodes [31]. In that matter, the definition of the anode is based on the electrode through which conventional current (positive charge) flows into the device from the external circuit, while an electrode through which conventional current flows out of the device is known as cathode. That is to say, if the direction of the current through the electrodes is reversed, as an example of a rechargeable battery when being charged, the naming of the electrodes is also reversed.

As previously mentioned, the material is among the important factors in the performance of electrochemical methods [32]. In the market, there are already many types of electrode materials used for wastewater treatment, such as; aluminum (Al), graphite (Gr), titanium (Ti), as well as iron (Fe). There are two main features for an electrode material, which are; being electronically conductive, and having the capacity to interact with the molecule.

Electrochemical methods for wastewater treatment include electrocoagulation-electro flocculation and electroflotation [23], electro-reduction [33], direct and, indirect electrooxidation using redox mediators, and hydrogen peroxide [34], and photo-assisted electrochemical methods like photoelectron-Fenton and photo-electrocatalysis [35]. Electrocoagulation (EC) based on the in-situ production of coagulants from a soluble anode material such as iron and/or aluminum that is oxidized due to the applied current has piqued attention among these approaches.

The general treatment mechanism starts with connecting the EC system to a power source. Then immediately an oxidation process starts to occur in the anode, the process that makes the electrode electrochemically corroded, meanwhile the passivation process occurs in the cathode. The process generates $M_{\left(aq\right)}^{3+}$ and *OH*^−^ ions which react to form various hydroxo monomeric and polymeric species. However, the formation of various hydroxo monomeric and polymeric species is highly dependent on the pH range. From the reaction of the two ions, M(OH)_3_ is from according to complex precipitation kinetics. Lastly, the pollutants dissolved and suspended in the wastewater are then adsorbed by the coagulants and then removed by either sedimentation or flotation [36].

However, the performance of electrochemical treatment approaches for wastewater treatment can be highly influenced by the type of electrodes used, arrangement of the electrodes as well as the type or characteristics of the wastewater to be treated. However, the potential effect of electrode polarity on the removal of pollutants from poultry slaughterhouse wastewater is still scarce.

In the current work, the effect of the polarity direction on the removal of pollutants from poultry slaughterhouse wastewater is investigated based on Ti and Al electrode materials. The study is generally divided into two cases; the first case is based on the wastewater being subjected to the Ti (anode)-Al (cathode) arrangement, and case II is when the wastewater is subjected to the Al (anode)-Ti (cathode) arrangement. The two cases were designed to investigate whether the polarity direction for the selected electrode materials will affect the removal of pollutants or not.

## 2. Results and Discussion

### 2.1. Data Distribution in the Raw Wastewater

**Figure 1:**
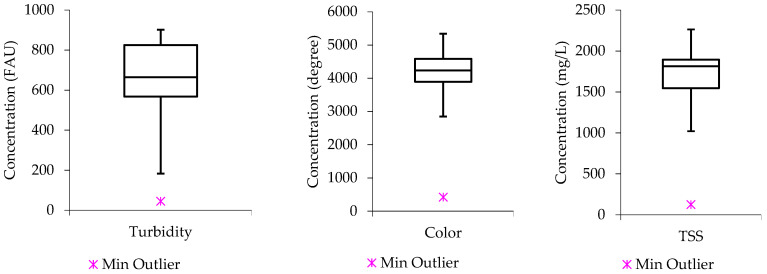
Raw wastewater boxplots from turbidity, color, and TSS.

Figure 1, shows that the selected water quality parameters had data distribution ranging from positive unsymmetrical nature to negative unsymmetrical as well as some potential equal distribution. For instance, the computed boxplot for turbidity is seen with the median approaching the lower quartile; a phenomenon that defines the data distribution to be positively skewed. In contrast to the turbidity data distribution, the color plot demonstrates that the series of concentration values had an equal distribution between high and low concentration values. While the TSS plot depicts that the series of concentration values has more low concentration values than high concentration values, with the median closer to the upper quartile, the data distribution is negatively skewed.

**Figure 2:**
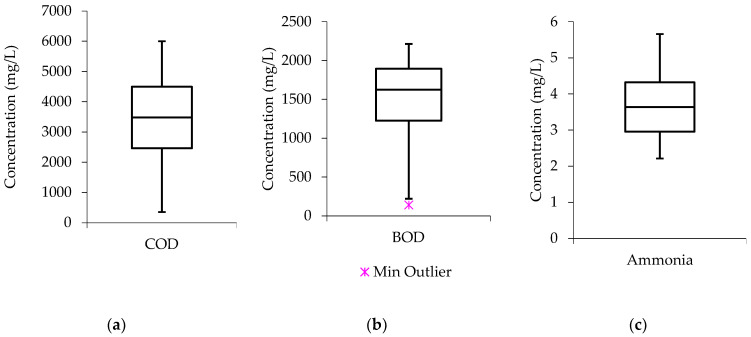
Raw wastewater boxplots (**a**) COD (**b**) BOD (**c**) ammonia.

Figure 2, shows that the COD boxplot has the median line closer to the middle, meaning that the data distribution is symmetric or normal. The median line in the BOD boxplot is closer to the upper quartile, indicating that the data distribution is “negatively skewed.” This suggests that low concentration values were more common in the BOD data than high concentration values. Similar, to the COD boxplot, the ammonia data distribution is symmetrical.

**Figure 3:**
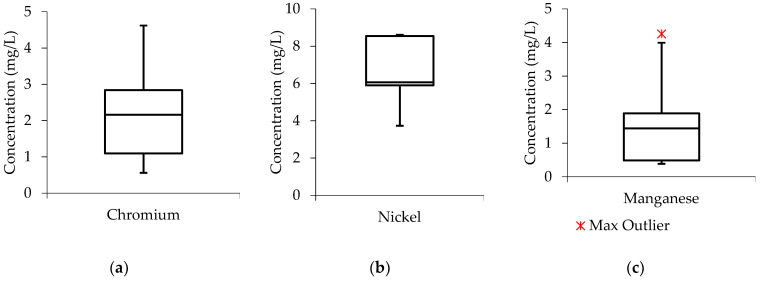
Raw wastewater boxplots (**a**) chromium (**b**) nickel (**c**) manganese.

The chromium and manganese datasets were more of negative skewness distribution as determined by the median lines in Figure 3; whereby, the medians can be observed to be closer to the upper quartile with a definition that the chromium and manganese data from raw wastewater constituted a higher frequency of low concentration values than the high concentration values. While the median line in the nickel boxplot is almost touching the lower quartile, this indicates that the water quality data has a larger frequency of high concentration values than low concentration values, a phenomenon known as “positive skewness.

### 2.2. Correlation among Parameters in the Raw Wastewater

Also, correlation matrices were developed to investigate the strength of relationships among the investigated water quality parameters in raw wastewater. From Table 1, a very strong correlation can be observed among turbidity, color, TSS, COD, and BOD with a correlation index ranging from 0.75 to 0.99. The highest correlation can be observed between color and turbidity with a correlation index of 0.99. The high correlation between color and turbidity can be linked to the fact that the more intensive the color is, the more light is absorbed and the turbidity appears to be higher than it is. This answers the question of why the real turbidity in a water sample is measured as scattered light. In that matter, relative to the general light reduction say to 0 Pt-Co by intensive colors dissolved in water, the final turbidity based on scattered light can then be exactly determined. Generally, the BOD to COD ratio serves as an indicator of the proportion of the organic matter that can be biologically degraded to total organic matter present in water. Therefore, the concentration levels or measures of COD concentration in water can be highly affected by the BOD concentration, a phenomenon that answers the high correlation between COD and BOD.

### 2.3. Analysis of the Treated Effluent

#### 2.3.1. Titanium (Anode) to Aluminium (Cathode) Electrode Arrangement (Ti-Al)

Table 2, shows that minimum and maximum concentrations of 0 FAU and 1.5 FAU were recorded from turbidity, respectively when the wastewater was subjected to the titanium (anode) to aluminium (cathode) electrode arrangement (Ti-Al). While 0.283 FAU was recorded as an average concentration. The turbidity average concentration is below the 5 FAU limit set by WHO for drinking water quality. Also, 0 Pt-Co and 0 mg/L were recorded as minimum concentration values for color and TSS, respectively. While, 59 Pt-Co and 5 mg/L were recorded as maximum concentration values for color and TSS, respectively. The average color concentration (29 Pt-Co) is beyond the recommended limit set for drinking water quality. However, the average TSS concentration is below the recommended limit.

In general, 0 mg/L was also recorded as the minimum concentration value for some other parameters such as ammonia, chromium, and nickel. This is to say that, in some of the experiments, the Ti-Al electrode arrangement was able to completely remove the aforementioned parameters.

An average COD concentration of 126.3 mg/L was achieved from the effluent treated by the Ti-Al electrode arrangement; a difference of 3232.7 mg/L from the raw wastewater. While an average BOD concentration of 15.95 mg/L was achieved when the wastewater was subjected to the Ti-Al electrode arrangement.

#### 2.3.2. Aluminium (Anode) to Titanium (Cathode) Electrode Arrangement (Al-Ti)

Table 3, shows that 0 FAU was recorded as a minimum, maximum, median, and average concentrations from turbidity when the wastewater was subjected to the aluminium (anode) to titanium (cathode) electrode arrangement (Al-Ti). That is to say that, unlike the Ti-Al electrode arrangement, the Al-Ti arrangement was able to completely remove turbidity from the wastewater, and 0 FAU turbidity was recorded from all the experiments sessions.

Also, 0 Pt-Co and 0 mg/L were recorded as minimum concentration values for color and TSS, respectively. While 59 Pt-Co was recorded as the maximum concentration value for the color. The average color concentration (15 Pt-Co) is also a bit lower than that was recorded from the Ti-Al electrode arrangement (29 Pt-Co). As for turbidity, 0 mg/L was also achieved as an average concentration for TSS when the wastewater was subjected to the Al-Ti electrode arrangement. Also, that is to say, that, unlike the Ti-Al electrode arrangement, the Al-Ti arrangement was able to completely remove TSS from the wastewater, and 0 mg/L of TSS was recorded from all the experiments sessions.

For some other parameters such as ammonia, chromium, and nickel, 0 mg/L was also recorded as the minimum concentration value. This means, at least once in the experiment sessions, the Al-Ti electrode arrangement was able to completely remove the above parameters.

An average COD concentration of 26.57 mg/L was achieved from the effluent treated by the Ti-Al electrode arrangement. The 26.57 mg/L is 4.8 times lower or 79% lower than the average COD concentration achieved when the wastewater was subjected to the Ti-Al electrode arrangement. While an average BOD concentration of 10.39 mg/L was achieved when the wastewater was subjected to the Ti-Al electrode arrangement; which is equivalent to 1.5 times lower or 34.9% lower than the average BOD concentration achieved when the wastewater was subjected to the Ti-Al electrode arrangement.

**Figure 4:**
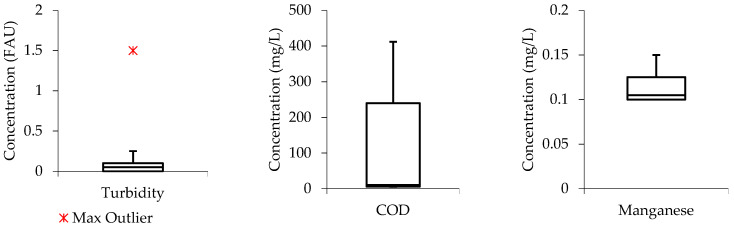
Boxplots from Ti-Al treated effluent.

To assess the distribution of data from the studied parameters in the treated effluents, box and whisker plots were also developed. In this case, the water quality parameters (turbidity, COD, and manganese) were selected as case studies. Figure 4, shows that the median line of the turbidity boxplot is closer to closer to the middle, indicating that the data distribution within the boxplot is symmetric or normal. While, the general boxplot touched the zero line, meaning that the turbidity concentration values were relatively low in the treated effluent when the wastewater was subjected to the Ti-Al electrode arrangement. For the COD and manganese boxplots, the median line can be observed to be closer to the lower quartile meaning that the water quality data constitute a higher frequency of more high concentration values than the low concentration values, a phenomenon that can be termed as “positive skewness”. Meaning that the water quality data distribution within the boxplot constituted a higher frequency of more high concentration values than the low concentration values, a phenomenon that can be termed as “positive skewness”.

**Figure 5:**
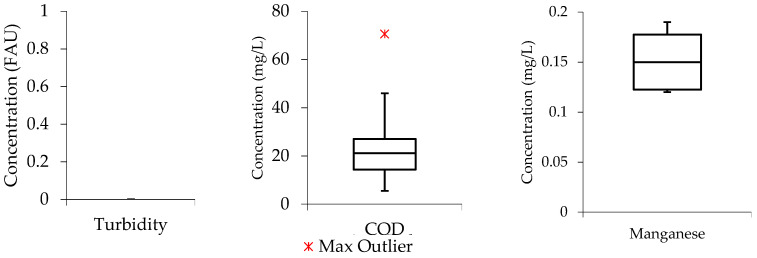
Boxplots from Ti-Al treated effluent.

Figure 5, shows that the turbidity boxplot is empty with an indication that the Al-Ti electrode arrangement achieved 100% removal efficiency from turbidity. The COD and manganese median lines are observed to be closer to the middle of the boxplots indicating that the data distribution is symmetric or normal.

### 2.4. Removal Efficiencies

#### 2.4.1. Hydraulic Retention Time—20 min

**Figure 6:**
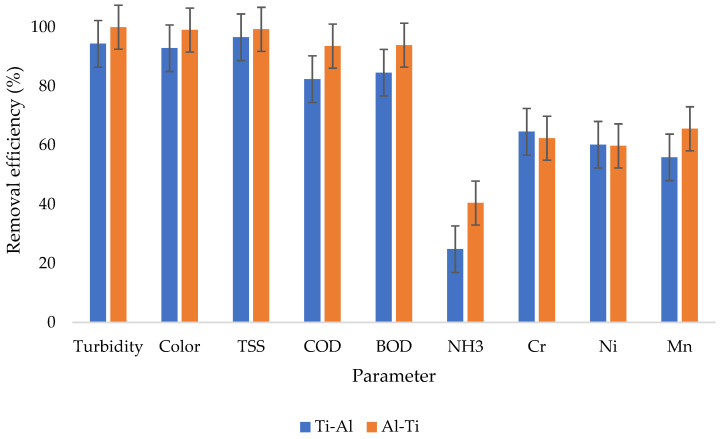
Removal efficiencies from 20 min retention time.

Figure 6 presents the removal efficiencies from 20 min retention time; it can be observed that despite a close tie in terms of removal efficiencies between the two investigated treatment approaches, there are still some slight discrepancies indicating that the Al-Ti electrode arrangement outperformed the Ti-Al electrode arrangement for most of the water quality parameters except for chromium and nickel; whereby, the removal efficiencies Ti-Al polarity had a little higher removal efficiencies from chromium and nickel.

Moreover, a *t*-test (Two-Sample Assuming Equal Variances) analysis was performed to compare the concentrations of each water quality parameter from the two treatment approaches. From the analysis results, it was observed that the *p*-values for turbidity (0.0089), TSS (0.0144), COD (0.0424), ammonia (0.0258), manganese (0.0226) color (0.0094), BOD (0.0114), chromium (0.0246) were less than 0.05; a phenomenon that rejects the null hypothesis, and justify that the data means were different. However, for nickel; the *p*-value (0.1685) was higher than 0.05, failing to reject the null hypothesis.

#### 2.4.2. Hydraulic Retention Time—40 min

**Figure 7:**
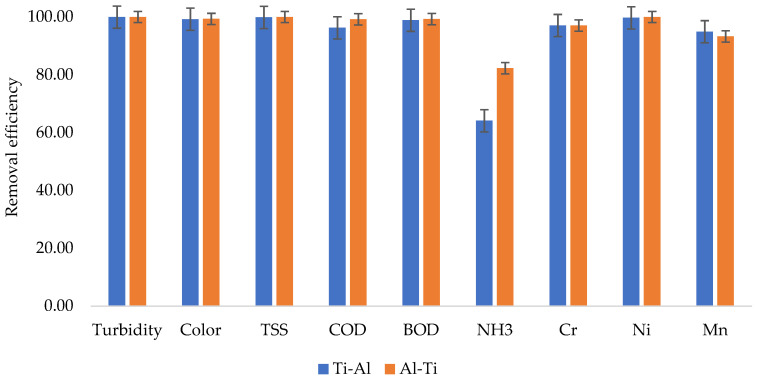
Removal efficiencies from 40 min retention time.

Figure 7 presents the results from 40 min retention time; shows that the two electrode arrangements were highly effective in terms of pollutants removal, with up to 100% removal efficiency achieved from turbidity when the wastewater was subjected to the Al-Ti electrode arrangement, as well as up to 99.95% removal efficiency when the wastewater was subjected to the Ti-Al electrode combination. Very high removal efficiencies can also be observed from color, TSS, COD, BOD, Cr, Ni, and Mn. A little challenge in terms of removal efficiency for both electrode arrangements can be observed, marking the lowest removal efficiencies from the investigation. In the literature, the EC treatment methods have also been observed to be highly effective in the removal of pollutants such as COD in other types of wastewaters. For instance, according to the study conducted by Chopra and Sharma [37], which investigated the effect of EC purification on the COD removal from biologically treated municipal wastewater, up to 85.8% removal efficiency was achieved. Generally, from the removal efficiencies, the Al-Ti electrode arrangement performed slightly higher than the Ti-Al arrangement, except for manganese.

From *t*-test (Two-Sample Assuming Equal Variances) analysis results, the *p*-values for turbidity (0.0136), TSS (0.0208), COD (0.0223), ammonia (0.0119), and manganese (0.0399) were less than 0.05; a phenomenon that rejects the null hypothesis, and justify that the data means were different. However, for color, BOD, chromium, and nickel; the *p*-values were higher than 0.05, failing to reject the null hypothesis.

### 2.5. Percent Compliance

**Figure 8:**
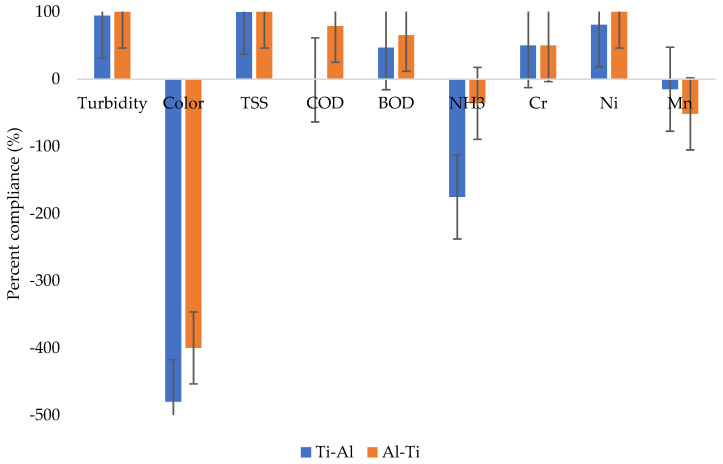
Percent compliance from Ti-Al and Al-Ti treatment systems.

Figure 8 provides a summary of the percent compliance of the studied water quality parameters based on drinking water quality standards. The parameters above zero line (positive values) are the ones complying with the drinking standards, while those below zero (negative values) are the ones not complying with the standards. The general trends show that the electrode combinations had a similar pattern in terms of pollutants removal and compliance to the drinking water quality standards. Although the Ali-Ti electrode combination can be observed to show better compliance than the Ti-Al electrode arrangement. The highest compliance is seen from the combination of Al-Ti electrode combination and TSS with 100% compliance. The lowest compliance can be observed from the combination of Ti-Al electrode combination and color, with compliance of −480%. In general, turbidity, TSS, COD, BOD, Cr, and Ni were within the compliance levels, while color, ammonia, and manganese were below the compliance levels.

## 3. Materials and Methods

### 3.1. Case Study, Water Samples, and Analytical Methods

The research work used samples collected from one of the biggest poultry farms in Central Asia located in Izhevsk village, in Kazakhstan. A discrete sampling approach was used to collect wastewater samples in 5 L plastic bottles. To preserve the natural condition of the samples, had to be stored at 4 °C before being subjected to transportation, analysis, and treatment. Generally, nine physical and chemical water quality parameters were considered in this research work, namely; turbidity, color, total suspended solids (TSS), biochemical oxygen demand (BOD), chemical oxygen demand (COD), ammonia (NH4)-nutrient, as well as potentially toxic elements; chromium (Cr), nickel (Ni), and manganese (Mn).

After transporting the samples to the lab, were then analyzed to check the quality characteristics of the raw wastewater (Table 4), and after treatment samples were again collected to check the quality of the treated effluent. During the analysis, several scientific procedures, test kits, and reagents were used to assess the quality of the water. The ammonia concentration levels in the samples were determined using the multiparameter 7500 Photometer (Palintest, CO, USA), with standard reagents as well as the test kits. The potentially toxic elements (chromium (Cr), nickel (Ni), manganese (Mn)) in the water samples were determined using the Atomic Absorption Spectrometry (Analytik Jena, Upland, CA, USA). In summary, the analysis of all the investigated water quality parameters was accomplished following the recommendations in the APHA Standard Methods for the Examination of Water and Wastewater [38].

### 3.2. Experimental Setup

The EC experiments were carried out at 22 ± 1 °C, while the wastewater within the reactor (1.7 L) was stirred continuously by a magnetic stir bar. The electrochemical reactor used in this study had a dimension of 15 × 13 × 11 cm^3^. The ranges of power supply were 0 to 50 V, and 0 to 10 A, with 24 V and 5.5 A being the average values (Table 5). Two main cases were investigated based on electrode materials and arrangement (polarity) (Figure 1); in the first case titanium electrode (10.8 × 11.8 × 0.2 cm^3^) was used as anode and aluminium electrode (10.8 × 11.8 × 0.2 cm^3^) was used as cathode. In the second case, the aluminium electrode was used as an anode and the titanium electrode was as a cathode. Also, in the study, anode and cathode electrodes were placed parallel with 2 cm.

**Figure 9:**
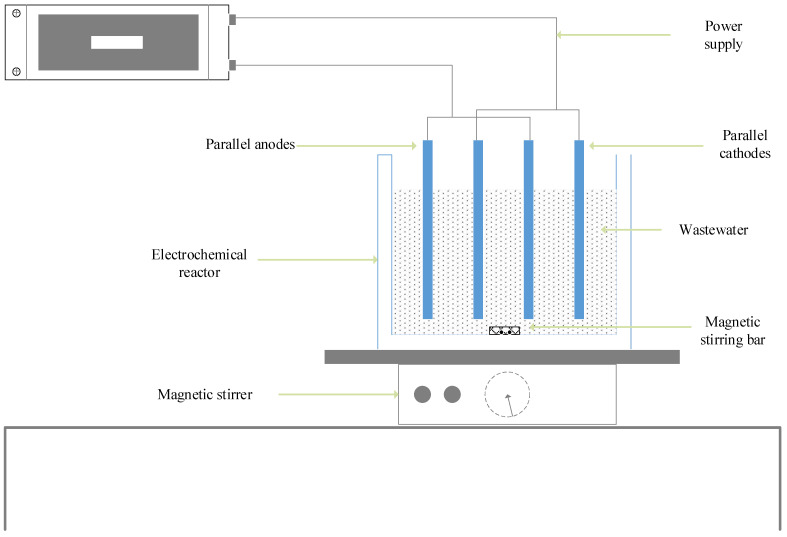
Experimental setup.

Figure 9 provides a summary of the general electrochemical process used to investigate the potential influence of polarity direction on the removal of pollutants from poultry slaughterhouse wastewater.

### 3.3. Statistical Analysis

#### 3.3.1. Removal Efficiency Analysis

Both raw wastewater and purified effluent were subjected to statistical analysis, including the removal efficiency computations based on the electrode materials (aluminium and titanium) and their arrangements. Percent removal analysis was used to investigate the performance of the studied electrochemical systems based on the polarity arrangements. In summary, the approach used to calculate the treatment efficiencies is based on Equation (1).

(1) $$T_{e}\left(\%\right)=\frac{C_{r}-C_{t}}{C_{r}}\times 100$$

whereby; *T_e_* stands for the removal efficiency; *C_r_* stands for the concentration in the raw wastewater and *C_t_* stands for the concentration after treatment.

The final effluent was also analyzed for conformity with various drinking water quality requirements. The approach utilized for compliance computation is summarized in Equation (2).

(2) $$C_{p}\left(\%\right)=\left(\frac{S_{i}-C_{i}}{S_{i}}\right)\times 100$$

whereby;

$C_{p}$, percent compliance,
$S_{i},$ the recommended standard for an i^th^ parameter,
$C_{i},\;\mathrm{the}\;$ concentration of the i^th^ parameter.

#### 3.3.2. Relationship Analysis

Apart from the removal efficiencies, the strength of the relationships among the selected water quality parameters was investigated based on the correlation coefficients. Investigation of the relationships among the parameters plays an important role in identifying the potential interaction between one parameter and another as well as data quality check. Statistically, when two or more parameters are characterized by high correlation as defined by the correlation indices or coefficients, portrays that there is a strong relationship between or among them. While a small correlation coefficient portrays that the parameters are hardly related.

Moreover, *t*-test (Two-Sample Assuming Equal Variances) was used to determine if there is a significant difference between the means of two groups of each parameter as determined by the two treatment approaches. The *t*-value expresses the magnitude of the difference in terms of the variation in the data. Therefore, the bigger the value of T, the more evidence there is that the null hypothesis is false.

#### 3.3.3. Data Distribution Analysis

It was also of significant importance to investigate the nature of data distribution of the water quality parameters; this was achieved with the help of vertical box and whisker plots. The presentation of water quality analysis based on average concentration values alone can sometimes be highly misleading; as some of the averaged values might have been affected by outliers that are huge enough to distort the real representation of water quality. Therefore, box and whisker plots provide a more convenient way of summarizing the results of the water quality analysis; whereby, the numerical data distribution and skewness are displayed in a form of a graph. More specifically, the plots are segmented into data quartiles and averages. The boxplots with maximum or minimum outliers portray that the values are substantially greater or lower than the other values in a data collection, or a value that is outside the data set. Moreover, the data distribution check is an important aspect of water quality as it gives an understanding of the pollution levels in the studied samples.

## 4. Conclusions

The potential influence of polarity direction on the removal of pollutants from poultry slaughterhouse wastewater using titanium (Ti) and aluminium (Al) electrode materials has been investigated. Two main cases were covered in this study; case number one, the titanium electrode is used as anode and the aluminium electrode is used as cathode. Case number two, the aluminium electrode is used as an anode, and the titanium electrode is as a cathode. Nine physicochemical water quality parameters (turbidity, color, total suspended solids (TSS), biochemical oxygen demand (BOD), chemical oxygen demand (COD), ammonia (NH4)-nutrient, as well as potentially toxic elements; chromium (Cr), nickel (Ni), and manganese (Mn)) were used in the investigation. In general, a high dispersion in terms of concentrations in the raw wastewater was observed; the phenomenon can be highly linked to the fact that in some days the less polluted sources of wastewater in the slaughterhouse generate more wastewater leading to dilution. From the analysis results, it was observed that the two electrode arrangements were highly effective in terms of pollutants removal, with up to 100% removal efficiency achieved from turbidity when the wastewater was subjected to the Al-Ti electrode arrangement, as well as up to 99.95% removal efficiency when the wastewater was subjected to the Ti-Al electrode combination. Very high removal efficiencies were also observed from color, TSS, COD, BOD, Cr, Ni, and Mn. A little challenge in terms of removal efficiency for both electrode arrangements can be observed, marking the lowest removal efficiencies from the investigation. Generally, from the removal efficiencies, the Al-Ti electrode arrangement performed slightly higher than the Ti-Al arrangement, except for manganese. Based on compliance, was observed from the of Al-Ti electrode combination and TSS with 100% compliance. The lowest compliance can be observed from the combination of Ti-Al electrode combination and color, with compliance of -480%. In general, turbidity, TSS, COD, BOD, Cr, and Ni were within the compliance levels, while color, ammonia, and manganese were below the compliance levels. The results from this study revealed further the potential of aluminium and titanium electrode materials for the purification of poultry slaughterhouse wastewater; with a high potential of performing impressively with other types of wastewaters.

## Figures and Tables

**Table 1:** Correlation of turbidity, color, TSS, COD, and BOD in the raw wastewater.

	Turbidity	Color	TSS	COD	BOD
Turbidity	1				
Color	0.99	1			
TSS	0.87	0.88	1		
COD	0.77	0.75	0.88	1	
BOD	0.80	0.85	0.80	0.81	1

**Table 2:** Effluent quality characteristics from titanium (anode) to aluminium (cathode) electrode arrangement.

Parameter	Min	Max	Median	Mean	STD
Turbidity	0	1.5	0.05	0.283	0.546
Color	0	59	32	29	18.102
TSS	0	5	1.5	2	1.633
COD	4.8	412	10.05	126.333	170.225
BOD	4.68	31.2	15.315	15.945	9.441
Ammonia	0	2.24	1.35	1.377	0.763
Chromium	0	0.1	0.05	0.05	0.05
Nickel	0	0.06	0	0.019	0.027
Manganese	0.1	0.15	0.105	0.115	0.019

**Table 3:** Effluent quality characteristics from aluminium (anode) to titanium (cathode) electrode arrangements.

Parameter	Min	Max	Median	Mean	STD
Turbidity	0	0	0	0	0
Color	0	35	19	15	12.298
TSS	0	0	0	0	0
COD	5.5	70.6	21.15	26.567	20.997
BOD	8.08	13	10.21	10.393	1.790
Ammonia	0	1.14	0.9	0.680	0.503
Chromium	0	0.1	0.05	0.050	0.050
Nickel	0	0	0	0	0
Manganese	0.12	0.19	0.15	0.152	0.029

**Table 4:** General characteristics of the poultry slaughterhouse wastewater (no = 12).

Parameter	Min	Max	Median	Mean	STD	Guideline	Agency	Unit
Turbidity	45.4	902	664	600.88	301.505	5	WHO	FAU *
Color	422	5340	4235	3694.4	1705.302	5	WHO	degree
TSS	126	2264	1814	1528.8	738.1153	500	KZ	mg/L
COD	358	5998	3480	3359	1901.393	125	KZ	mg/L
BOD	139.6	2214	1625	1419.52	717.577	30	KZ	mg/L
Ammonium	2.21	5.66	4.03	3.836	1.208	0.5	KZ	mg/L
Manganese	0.387	4.26	1.44	1.6934	1.403	0.1	WHO	mg/L
Nickel	3.73	8.61	6.06	6.5684	1.834	0.1	WHO	mg/L
Chromium	0.56	4.62	2.16	2.255	1.426	0.1	WHO	mg/L

* FAU = Formazin Attenuation Units.

**Table 5:** General technical specifications.

Parameter	Value	Unit
Initial water temperature	5–10	°C
Potential (voltage)	24	V
Average current density	5.5	A
Average power	132	W
Hydraulic retention time	40	min

## References

[1] Ronaldo R. (2020). Measuring the performance of poultry business through effective supply chain management skills. Uncertain Supply Chain Manag..

[2] Meiramkulova K., Jakupova Z., Orynbekov D., Tashenov E., Kydyrbekova A., Mkilima T., Inglezakis V.J. (2020). Evaluation of Electrochemical Methods for Poultry Slaughterhouse Wastewater Treatment. Sustainability.

[3] Aziz H., Puat N., Alazaiza M., Hung Y.-T. (2018). Poultry Slaughterhouse Wastewater Treatment Using Submerged Fibers in an Attached Growth Sequential Batch Reactor. Int. J. Environ. Res. Public Health.

[4] Basitere M., Rinquest Z., Njoya M., Sheldon M.S., Ntwampe S.K.O. (2017). Treatment of poultry slaughterhouse wastewater using a static granular bed reactor (SGBR) coupled with ultrafiltration (UF) membrane system. Water Sci. Technol..

[5] Rajab A.R., Salim M.R., Sohaili J., Anuar A.N., Salmiati, Lakkaboyana S.K. (2017). Performance of integrated anaerobic/aerobic sequencing batch reactor treating poultry slaughterhouse wastewater. Chem. Eng. J..

[6] Meiramkulova K., Devrishov D., Marzanov N., Marzanova S., Kydyrbekova A., Uryumtseva T., Tastanova L., Mkilima T. (2020). Performance of Graphite and Titanium as Cathode Electrode Materials on Poultry Slaughterhouse Wastewater Treatment. Materials.

[7] Terán Hilares R., Atoche-Garay D.F., Pinto Pagaza D.A., Ahmed M.A., Colina Andrade G.J., Santos J.C. (2021). Promising physicochemical technologies for poultry slaughterhouse wastewater treatment: A critical review. J. Environ. Chem. Eng..

[8] Delforno T.P., Lacerda Júnior G.V., Noronha M.F., Sakamoto I.K., Varesche M.B.A., Oliveira V.M. (2017). Microbial diversity of a full-scale UASB reactor applied to poultry slaughterhouse wastewater treatment: Integration of 16S rRNA gene amplicon and shotgun metagenomic sequencing. Microbiologyopen.

[9] Meiramkulova K., Zorpas A.A., Orynbekov D., Zhumagulov M., Saspugayeva G., Kydyrbekova A., Mkilima T., Inglezakis V.J. (2020). The Effect of Scale on the Performance of an Integrated Poultry Slaughterhouse Wastewater Treatment Process. Sustainability.

[10] Baker B.R., Mohamed R., Al-Gheethi A., Aziz H.A. (2021). Advanced technologies for poultry slaughterhouse wastewater treatment: A systematic review. J. Dispers. Sci. Technol..

[11] Njoya M., Basitere M., Ntwampe S.K.O., Lim J.W. (2021). Performance evaluation and kinetic modeling of down-flow high-rate anaerobic bioreactors for poultry slaughterhouse wastewater treatment. Environ. Sci. Pollut. Res..

[12] Fatima F., Du H., Kommalapati R.R. (2021). Treatment of Poultry Slaughterhouse Wastewater with Membrane Technologies: A Review. Water.

[13] Paulista L.O., Presumido P.H., Theodoro J.D.P., Pinheiro A.L.N. (2018). Efficiency analysis of the electrocoagulation and electroflotation treatment of poultry slaughterhouse wastewater using aluminum and graphite anodes. Environ. Sci. Pollut. Res..

[14] Ardestani F., Abbasi M. (2019). Poultry Slaughterhouse Wastewater Treatment Using Anaerobic Fluid Bed Reactor and Aerobic Mobile-Bed Biological Reactor. Int. J. Eng..

[15] Narayanan C.M., Narayan V. (2019). Biological wastewater treatment and bioreactor design: A review. Sustain. Environ. Res..

[16] Lakatos G. (2015). Biological Wastewater Treatment. Principles of Membrane Bioreactors for Wastewater Treatment.

[17] Xu S., Wu X., Lu H. (2021). Overlooked nitrogen-cycling microorganisms in biological wastewater treatment. Front. Environ. Sci. Eng..

[18] Edwards J.K., Heath A.W. (2011). Biological Treatments. A Consumer’s Guide to Mental Health Services.

[19] Chollom M.N., Rathilal S., Swalaha F.M., Bakare B.F., Tetteh E.K. (2019). Anaerobic Treatment of Slaughterhouse Wastewater: Evaluating Operating Conditions. Proceedings of the WIT Transactions on Ecology and the Environment, Ashurst, New Forest, UK, 3–5 October 2019.

[20] Amin M., Rafiei N., Taheri E. (2016). Treatment of slaughterhouse wastewater in an upflow anaerobic sludge blanket reactor: Sludge characteristics. Int. J. Environ. Health Eng..

[21] Trishitman D., Cassano A., Basile A., Rastogi N.K. (2020). Reverse osmosis for industrial wastewater treatment. Current Trends and Future Developments on (Bio-) Membranes.

[22] Patel D., Mudgal A., Patel V., Patel J. (2021). Water desalination and wastewater reuse using integrated reverse osmosis and forward osmosis system. IOP Conf. Ser. Mater. Sci. Eng..

[23] Eryuruk K., Tezcan Un U., Bakır Ogutveren U. (2018). Electrochemical treatment of wastewaters from poultry slaughtering and processing by using iron electrodes. J. Clean. Prod..

[24] Asselin M., Drogui P., Benmoussa H., Blais J.-F. (2008). Effectiveness of electrocoagulation process in removing organic compounds from slaughterhouse wastewater using monopolar and bipolar electrolytic cells. Chemosphere.

[25] Ngobeni P.V., Basitere M., Thole A. (2021). Treatment of poultry slaughterhouse wastewater using electrocoagulation: A review. Water Pract. Technol..

[26] Sharma S., Simsek H. (2020). Sugar beet industry process wastewater treatment using electrochemical methods and optimization of parameters using response surface methodology. Chemosphere.

[27] Davarnejad R., Nikseresht M. (2016). Dairy wastewater treatment using an electrochemical method: Experimental and statistical study. J. Electroanal. Chem..

[28] Hoang T.L., Luu T.L. (2020). Fabrication of textile wastewater treatment block unit using electrochemical method. Desalin. Water Treat..

[29] Tien T.T., Luu T. (2019). Le Electrooxidation of tannery wastewater with continuous flow system: Role of electrode materials. Environ. Eng. Res..

[30] Al-Barakat H.S., Matloub F.K., Ajjam S.K., Al-Hattab T.A. (2020). Modeling and Simulation of Wastewater Electrocoagulation Reactor. IOP Conf. Ser. Mater. Sci. Eng..

[31] Bitenc J., Lindahl N., Vizintin A., Abdelhamid M.E., Dominko R., Johansson P. (2020). Concept and electrochemical mechanism of an Al metal anode—Organic cathode battery. Energy Storage Mater..

[32] Salazar-Banda G.R., Santos G.d.O.S., Gonzaga I.M.D., Dória A.R., Eguiluz K.I.B. (2021). Developments in electrode materials for wastewater treatment. Curr. Opin. Electrochem..

[33] Peng H., Leng Y., Cheng Q., Shang Q., Shu J., Guo J. (2019). Efficient removal of hexavalent chromium from wastewater with electro-reduction. Processes.

[34] Rethinam A.J., Kennedy C.J. (2004). Indirect electrooxidation of crotyl and cinnamyl alcohol using a Ni(OH)_2_ electrode. J. Appl. Electrochem..

[35] Yu F., Wang Y., Ma H., Dong G. (2018). Enhancing the yield of hydrogen peroxide and phenol degradation via a synergistic effect of photoelectrocatalysis using a g-C_3_N_4_/ACF electrode. Int. J. Hydrogen Energy.

[36] Kabdaşlı I., Arslan-Alaton I., Ölmez-Hancı T., Tünay O. (2012). Electrocoagulation applications for industrial wastewaters: A critical review. Environ. Technol. Rev..

[37] Chopra A.K., Sharma A.K. (2015). Effect of electrochemical treatment on the COD removal from biologically treated municipal wastewater. Desalin. Water Treat..

[38] Hasanah U., Mulyati A.H., Sutanto, Widiastuti D., Warnasih S., Syahputri Y., Panji T. (2020). Development of COD (Chemical Oxygen Demand) Analysis Method in Waste Water Using UV-VIS Spectrophotometer. J. Sci. Innovare.

